# Characterization of Potting Epoxy Resins Performance Parameters Based on a Viscoelastic Constitutive Model

**DOI:** 10.3390/polym16070930

**Published:** 2024-03-28

**Authors:** Lin Yang, Anxin Ding, Mi Xu, Yuefang Li, Xianhang Zhao, Jingxuan Peng, Xiang Li

**Affiliations:** 1 School of Materials Science and Engineering, Wuhan University of Technology, Wuhan 430070, China; 317302@whut.edu.cn (L.Y.); axding@whut.edu.cn (A.D.); mix0121@whut.edu.cn (M.X.); 18986980044@163.com (J.P.); 2 Institute of Electronic Engineering, China Academy of Engineering Physics, Mianyang 621999, China; yuefangli_caep@163.com (Y.L.); xhz@mail.nwpu.edu.cn (X.Z.)

**Keywords:** epoxy resin, residual stress, viscoelastic properties, bulk modulus, shear modulus

## Abstract

To describe the evolution of residual stresses in epoxy resin during the curing process, a more detailed characterization of its viscoelastic properties is necessary. In this study, we have devised a simplified apparatus for assessing the viscoelastic properties of epoxy resin. This apparatus employs a confining cylinder to restrict the circumferential and radial deformations of the material. Following the application of load by the testing machine, the epoxy resin sample gradually reduces the gap between its surface and the inner wall of the confining cylinder, ultimately achieving full contact and establishing a continuous interface. By recording the circumferential stress–strain on the outer surface of the confining cylinder, we can deduce the variations in material bulk and shear moduli with time. This characterization spans eight temperature points surrounding the glass transition temperature, revealing the bulk and shear relaxation moduli of the epoxy resin. Throughout the experiments, the epoxy resin’s viscoelastic response demonstrated a pronounced time-temperature dependency. Below the glass transition temperature, the stress relaxation response progressively accelerated with increasing temperature, while beyond the glass transition temperature, the stress relaxation time underwent a substantial reduction. By applying the time-temperature superposition principle, it is possible to construct the relaxation master curves for the bulk and shear moduli of the epoxy resin. By fitting the data, we can obtain expressions for the constitutive model describing the viscoelastic behavior of the epoxy resin. In order to validate the reliability of the test results, a uniaxial tensile relaxation test was conducted on the epoxy resin casting body. The results show good agreement between the obtained uniaxial relaxation modulus curves and those derived from the bulk and shear relaxation modulus equations, confirming the validity of both the device design and the testing methodology.

## 1. Introduction

Potting compounds play a pivotal role in encapsulating semiconductor chips and closed-circuit board components, as well as wires and solder joints. Their primary function is to provide robust protection against mechanical damage, moisture, chemicals, and dust, thereby enhancing the durability and stability of the chip and circuit boards. This protection is particularly crucial in demanding environments characterized by elevated temperatures, humidity, and vibrations [1,2]. Commonly utilized potting materials predominantly consist of various polymer compositions, such as epoxy resin, polyurethane, polyimide, and so on. Among them, epoxy resin holds a prominent position in electronic packaging and related industries due to its excellent mechanical properties, commendable corrosion resistance, and other inherent advantages [3].

Attributable to factors like curing shrinkage during chemical reactions and a mismatch of thermal expansion coefficients between the resin and the substrate, epoxy resin generates residual stresses during curing. Resulting in material property degradation, it imparts a discernible influence on structural performance and potentially leads to device obsolescence [4,5,6]. In addition, epoxy resin is commonly used as the matrix of composite materials, and its related properties can affect the mechanical performance of composites. Hence, it is imperative to investigate the evolution of residual stresses in polymers during the curing process, analyze the influencing factors of cured residual stresses, and subsequently optimize the process conditions to minimize the adverse effects of these cured stresses effectively [7].

Chunyan Qu et al. [8] utilized a thin-film stress analyzer to investigate changes in thermal stress within the modified epoxy system during the curing process and employed Stoney’s equation to calculate the curing stress within the system. The analysis concluded that the residual stress value of the modified epoxy resin system during the curing process was less than that of the pure epoxy. Employing an optimization algorithm through inverse analysis, Zafar Namazian et al. [9] optimized the thermal curing cycle to minimize exothermic peaks and temperature gradients during the curing process, thereby mitigating internal residual stresses. In contrast, establishing a constitutive model for the curing process of the material and employing theoretical or numerical methods to accurately quantify the influencing factors of curing stresses is a more common and reliable approach.

Polymers exhibit pronounced viscoelastic behavior, especially under elevated temperature conditions. However, owing to the intricacies of the viscoelastic constitutive model, which demands numerous parameters and poses testing challenges, simplified viscoelastic models are frequently employed, such as the linear elasticity model and the path-dependent model. In the rapidly evolving landscape of technology, particularly in the field of electronics engineering where encapsulation technology plays a pivotal role, given the escalating demands and the continuous evolution of technology, considering the influence of viscoelasticity during the curing process becomes imperative for a more accurate analysis of changes in residual stress during the epoxy resin curing process. A close relationship exists between viscoelastic properties and curing stress, wherein the intensity and duration of the curing stress depend on the properties of the viscoelastic material. Epoxy resins exhibiting viscoelasticity do not deform instantaneously after stress application but rather evolve gradually over time. This implies a time delay after subjecting the viscoelastic material to stress. The material undergoes gradual relaxation over time following viscoelastic deformation, resulting in a gradual weakening of internal stress [10].

A prevalent method for characterizing viscoelastic properties involves conducting uniaxial or shear relaxation modulus tests on materials [11,12]. Akshat Agha et al. [13] utilized a dynamic thermo-mechanical analyzer to conduct shear tests on a one-component epoxy-based thermosetting adhesive to investigate viscoelasticity and plastic deformation of the material during thermal curing, developing a viscoelastic model to predict residual stresses generated in the adhesive. Bàrbara Adrover-Monserrat et al. [14] determined the Prony series parameters of their viscoelastic model by conducting uniaxial relaxation tests on polyether block amide filaments, confirming the reliability of the model by rapid cyclic loading tests.

Nevertheless, current viscoelastic models allocate limited attention to the testing of bulk modulus. This limitation primarily stems from the requirements for high precision and the demand for specialized instrumentation [15], posing challenges in practical applications. As of now, a unified standard for bulk modulus testing has not been established, and commercially available testing equipment is scarce. Methods for determining the bulk modulus in the publicly reported literature can be broadly categorized into two groups. The first is the indirect testing method, where the corresponding bulk modulus value is derived by characterizing Young’s modulus, shear modulus, or Poisson’s ratio of the material [16,17]. Wolfgang Klimm et al. [18] performed a series of creep tests at different temperatures using biaxial strain measurements of the axial and transverse strains of the polymer matrix to obtain the uniaxial and shear creep compliance, respectively, and then derived the bulk relaxation modulus through a correlation equation. Toru Ikeda et al. [19] conducted stress relaxation tests on resins using the Digital Image Correlation Method (DICM) and Confocal Laser Scanning Microscopy to characterize the changes in material stress, axial strain, and transverse strain, obtained tensile relaxation curves and the Poisson’s ratio, and then applied the Prony series to fit the primary curves of the relaxation modulus. Subsequently, the material bulk modulus was determined based on the relationship derived from the Laplace transform.

An alternative is the direct test method, where the bulk modulus is calculated by applying hydrostatic pressure to the material and measuring bulk stress and strain according to the bulk modulus definition [20,21,22,23]. Koray Senol et al. [24] explored the applicability of the 3D digital image correlation technique in the context of material viscoelastic properties. The study focused on investigating the deformation of underwater objects within a large-thickness acrylic bending tube and analyzing the viscoelastic constitutive behavior of PVC foam plastics. The bulk modulus of foams of different densities under hydrostatic pressure was determined by recording the volume change in the specimen using two cameras perpendicular to the tube during loading in a universal testing machine. Hyun Seop Lee et al. [25] designed a compact chamber to test the viscoelastic properties of epoxy molding compounds, subjecting the experimental specimen to bulk modulus tests spanning temperatures from 25 °C to 235 °C. The discussion centered on the time-dependent changes in bulk modulus and their impact on warpage. Nevertheless, these test methods pose significant complexities, both in terms of equipment requirements and sample preparation.

Acknowledging the limitations of the aforementioned methods, this study focuses on E-44 epoxy resin as the research subject. A set of testing molds is designed, and this study proposes a straightforward experimental scheme to precisely measure the bulk stress and bulk strain of the epoxy resin. Subsequently, this study integrates experimental data with theoretical formulas to compute the bulk and shear modulus of the material. Leveraging the relaxation modulus curves of epoxy resin at different temperatures, this study applies the principles of time-temperature superposition and Maxwell’s formula to generate the relaxation master curves of epoxy resin bulk and shear modulus, providing expressions for the viscoelastic model. In conclusion, a uniaxial relaxation test is conducted to verify the reliability of the obtained results and the proposed viscoelastic model.

## 2. Materials and Test

### 2.1. Sample Preparation

We incorporated 20 g of curing agent I (D230 polyether amine) and 14 g of curing agent II (D400 polyether amine) into 100 g of E-44 epoxy resin (bisphenol A epoxy resin WSR6101), which were thoroughly blended and then put into a vacuum drying box (YIHENG, BPG-9106B, Shanghai, China) to remove air bubbles inside the mixture. The cylinder was affixed to the base plate using sealant tape with an even layer of release agent (TIANYING MIRROR WAX NO. 8) applied to the inside. The resin was poured into the defined cylinder, followed by another vacuum process to eliminate air bubbles. Subsequently, the mixture was placed in the oven (Dingyao Science and Technologies, DYCK-50D, Shenzhen, China) for curing. The curing process involved an initial curing at 55 °C for 3 h to achieve solidity. Following this, the cooled specimen was removed from the mold, and the curing continued in a 95 °C atmosphere at a constant temperature for an additional 3.5 h to ensure complete curing. After removing the cylinder, the specimen exhibited unconstrained volume expansion in the high-temperature environment. The dimensional stability before and after curing improved, facilitating subsequent cooperation with the cylinder.

### 2.2. DSC Testing

Determining the glass transition temperature (Tg) of the cured specimens was carried out using DSC (Perkin Elmer, DSC 4000, Waltham, MA, USA) to establish reference conditions for the subsequent selection of temperature points for the perimeter pressure test. The test temperature procedures involved initially maintaining a constant temperature of 30 °C for two minutes, followed by a temperature ramp at a rate of 10 °C/min up to 100 °C. Following a data analysis, the glass transition temperature of the sample was determined to be 75 °C. Temperature points for the subsequent viscoelastic test were selected within the temperature interval above and below the determined glass transition temperature. Table 1 presents the specific values of the selected test temperatures.

## 3. Viscoelastic Homeostasis Testing Device and Principle

### 3.1. Experimental Setup

**Figure 1:**
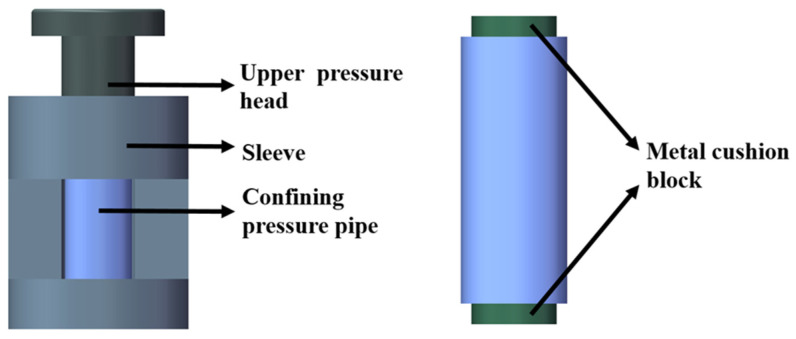
Material viscoelasticity testing mold.

In order to accurately and conveniently determine the bulk modulus and shear modulus of the epoxy resin, a test mold was designed, as shown in Figure 1. This apparatus mainly consists of an upper indenter, a sleeve, a confining cylinder, and two cylindrical metal blocks. The bottom of the upper indenter is designed with a groove that serves as a limiting feature, facilitating alignment with the two cylindrical metal blocks to uniformly apply the load to the specimen. The function of the sleeve is to ensure that the upper indenter and the defined cylinder do not tilt during the loading process. The cylinder, made of stainless steel, provides a sufficient constraint on the specimen, suppressing uneven deformation. The defined cylinder, in coordination with the upper and lower cylindrical metal blocks, forms triaxial pressure on the specimen, characterizing the viscoelastic behavior of epoxy resin under high stress levels [26]. Additionally, two high-temperature-resistant strain gauges (KYOWA, KFGS-3-120-C1-11 L1M3R, Aichi, Japan) were attached at the circumferential center position of the defined cylinder to avoid errors arising from variations in attachment locations.

### 3.2. Experimental Principles

To create smooth upper and lower surfaces, the fully cured specimens were cut, and the diameters and lengths of the specimens were measured and recorded. To ensure a good fit between the defined cylinder and the specimen, five different-sized cylinders were designed according to the specifications detailed in Table 2. Due to the different coefficients of thermal expansion between the specimen and the stainless steel tube, it was necessary to pre-match specimens and defined cylinders at different temperatures to achieve optimal fit. In the most ideal scenario, when the specimen and cylinder are stabilized at an appropriate ambient temperature, their gap is precisely equal to zero. At this point, applying a small load allows for the monitoring of the circumferential strain on the outer surface of the defined cylinder.

Utilizing a cylindrical coordinate system to illustrate the stress–strain relationship between the specimen and the defined cylinder, a universal testing machine was employed to apply a load to the specimen, and two extensometers were clamped on both sides of the mold to measure the axial deformation of the specimen, ensuring uniformity of axial deformation on both sides. The axial stress $\sigma_{ZZ}(r,\theta ,z)$ and axial strain $\varepsilon_{ZZ}(r,\theta ,z)$ of the specimen are represented by the following equation:

(1) $$\sigma_{zz}\left(r,\theta ,z\right)=\sigma_{i}=\frac{F}{\pi i^{2}}$$

(2) $$\varepsilon_{\mathrm{zz}}\left(r,\theta ,z\right)=\varepsilon_{i}=\frac{\bar{l}}{L}$$

where *F* is the load applied by the testing machine in the axial direction, *i* is the radius of the specimen, $\bar{l}$ is the average value of the deformation of the two extensometers, and *L* is the length of the specimen.

When subjected to axial stress, the cylinder restrains both radial and circumferential deformation of the specimen. The radial pressure exerted on the outer surface of the specimen is equal to that on the inner surface of the cylinder. The stress–displacement relationship at the interface between the inner wall of the cylinder and the outer surface of the specimen is expressed as follows: when $\chi_{r}(i)=\chi_{r}^{p}(i)>0$, $\sigma_{\mathrm{rr}}\left(i\right)=\sigma_{\mathrm{rr}}^{p}\left(i\right)=-\sigma$, and when $\chi_{r}(i)\leq 0$, the inner surface of the confining cylinder experiences no traction. The circumferential strain $\varepsilon_{c}$ is characterized by the average value of the double strain gauges on the outer surface of the defined cylinder. The radial components of the displacement and stress on the inner surface of the cylinder are deduced from the Lam’e solution [27] as follows:

(3) $$\sigma_{rr}^{p}\left(d\right)=-\sigma =-\frac{d^{2}-i^{2}}{2i^{2}}E^{p}\varepsilon_{c}$$

(4) $$u_{r}^{p}=\frac{\varepsilon_{c}}{2}\left[\left(1-\nu^{p}\right)i+\left(1+\nu^{p}\right)\frac{d^{2}}{i}\right]$$

where *d* is the outer diameter of the defined cylinder, *E*^p^ is the Young’s modulus of the cylinder, and $\nu^{p}$ is the Poisson’s ratio of the cylinder.

The specimen undergoes axisymmetric uniform deformation in the experiment, and its radial stress–strain is equal to the circumferential stress–strain, as shown in the following equation:

(5) $$\sigma_{\mathrm{rr}}=\left(r,\theta ,z\right)=\sigma_{\theta \theta }\left(r,\theta ,z\right)=-\sigma$$

(6) $$\varepsilon_{\mathrm{rr}}\left(r,\theta ,z\right)=\varepsilon_{\theta \theta }\left(r,\theta ,z\right)$$

In summary, the stress and strain of the specimen in three directions are as follows:

(7) $$\sigma_{\mathrm{rr}}\left(t\right)=\sigma_{\theta \theta }=-\frac{{\left(\frac{d}{i}\right)}^{2}-1}{2}E^{p}\varepsilon_{c}\left(t\right)$$

(8) $$\sigma_{\mathrm{zz}}\left(t\right)=\sigma_{i}\left(t\right)$$

(9) $$\varepsilon_{\mathrm{rr}}\left(t\right)=\varepsilon_{\theta \theta }=\frac{\varepsilon_{c}\left(t\right)}{2}\left[\left(1-\nu^{p}\right)+\left(1+\nu^{p}\right)\frac{d^{2}}{i^{2}}\right]$$

(10) $$\varepsilon_{\mathrm{zz}}(t)=\varepsilon_{i}(t)$$

Equations (7)–(10) allow for the characterization of the stress–strain of the material in the confining pressure state.

## 4. Results and Discussion

### 4.1. Viscoelastic Test

**Figure 2:**
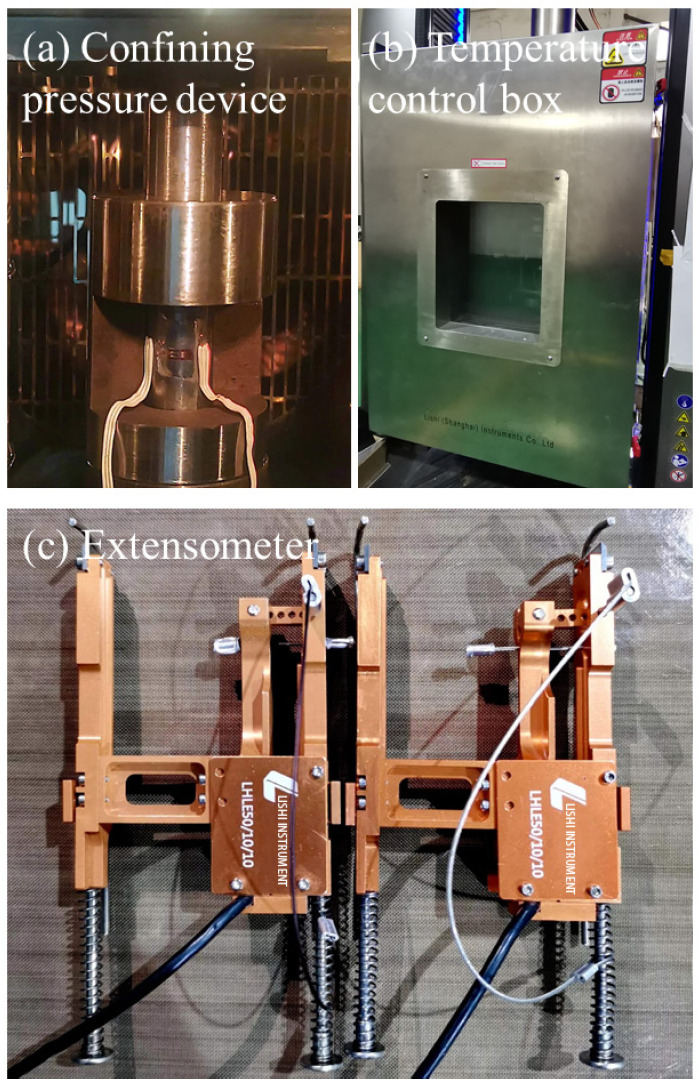
Viscoelastic test setup: (**a**) Confining pressure device; (**b**) Temperature control box; (**c**) Extensometer.

The universal testing machine is equipped with two control systems: one for displacement and load control and the other for temperature control, enabling a variety of temperature tests. The test apparatus is installed and secured as shown in Figure 2a, with extensometers (Force Test Coefficient LHLE50/10/10) clamped on the left and right sides, as illustrated in Figure 2b. Subsequently, the entire setup is transferred into the temperature-controlled chamber, as illustrated in Figure 2c.

We secured the specimen using displacement-controlled loading and securely fastened the strain gauge’s connection line with tape to prevent the connection line from shaking during the temperature control process, ensuring the accuracy of data collection. We set the desired testing temperature, and when the temperature reached the set value, we stabilized the temperature inside the chamber within ±0.5 °C of the set value. The cylinder functions as a thermal buffer, and the temperature of the specimen is not easily affected by the ambient temperature.

When the specimen is heated uniformly, an initial force of approximately 100 N is applied to the testing apparatus, followed by loading at a rate of 2 mm/min until reaching the target load. This target load is adjusted according to different experimental temperatures to achieve a circumferential strain of approximately 100 microstrains on the outer surface of the defined cylinder. Subsequently, the loading is stopped, the clamped end position is maintained, and the stress relaxation test is conducted to record the changes over time in the axial force F, axial deformation $l_{1}$ and $l_{2}$, and circumferential strain $\varepsilon_{c1}$ and $\varepsilon_{c2}$.

### 4.2. Bulk Modulus and Shear Modulus

The stresses and strains of the specimen in the three directions are calculated by the formulas described in the third section, and then the average stress $\sigma_{m}$ and the bulk strain $\varepsilon_{\mathrm{vol}}$ of the specimen are obtained based on the theoretical formulas, as shown in the following equations:

(11) $$\sigma_{m}=\frac{\sigma_{\mathrm{zz}}+\sigma_{\mathrm{rr}}+\sigma_{\theta \theta }}{3}$$

(12) $$\varepsilon_{\mathrm{vol}}=\varepsilon_{\mathrm{zz}}+\varepsilon_{\mathrm{rr}}+\varepsilon_{\theta \theta }$$

The calculated formulas for the bulk modulus and shear modulus of epoxy resin are derived as follows:

(13) $$K=\frac{\sigma_{m}}{\varepsilon_{\mathrm{Vol}}}$$

(14) $$G=\frac{\left(\sigma_{\mathrm{rr}}-\sigma_{m}\right)/2}{\varepsilon_{\mathrm{rr}}-\frac{1}{3}\varepsilon_{\mathrm{Vol}}}$$

This equation can describe the bulk modulus and shear modulus of resin under multiaxial stress, and it can also be used to study the micromechanical models of related composite materials, such as the Tsai-Wu [28] and Checkerboard [29] micromechanical models.

The viscoelastic bulk modulus and shear modulus measured at various temperatures of the specimen are summarized, and the results are presented in Table 3. At 40 °C, the initial bulk modulus and shear modulus of the epoxy resin can reach 4140 MPa and 1205 MPa. The initial bulk modulus and shear modulus decrease with an increase in temperature. The decrease is more pronounced as the temperature approaches the glass transition temperature of the specimen. At 85 °C, the initial bulk modulus of the specimen is reduced to 1862 MPa, while the initial shear modulus is reduced to 19 MPa.

**Figure 3:**
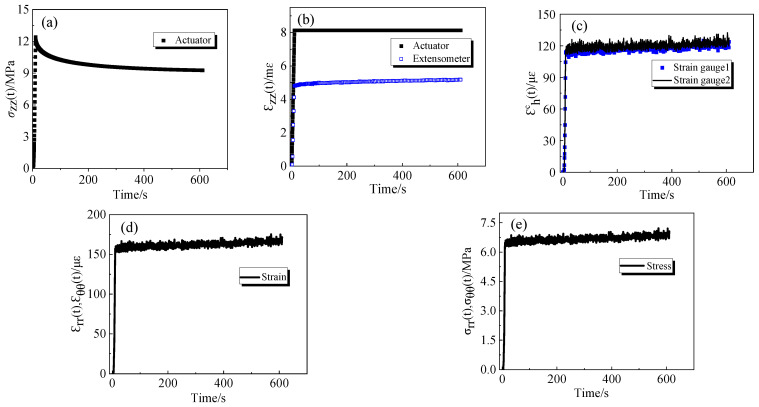
Viscoelastic test data plots of specimens at 70 °C: (**a**) change in axial stress of specimens with time; (**b**) change in axial strain recorded by the testing machine and axial strain measured by the extensometer with time; (**c**) change in annular and radial strains on the outer surface of cylinder with time; (**d**) change in annular and radial strains of the specimens with time; (**e**) change in annular and radial stress of the specimens with time.

The 70 °C test data were selected to analyze the viscoelastic response behavior of the epoxy resin, as shown in Figure 3. This temperature is in proximity to the glass transition temperature of the specimen, Tg = 75 °C. The increased motility of the molecules and chain segments of the resin enhances the stress relaxation phenomenon, as depicted in Figure 3a. Figure 3b illustrates the strain data automatically collected by the testing machine and the strain recorded by the extensometer over time. It reveals that the strain measured by the extensometer is smaller than that of the testing machine. The deformation measured by the testing machine contains the deformation of the parts of the testing machine itself and the deformation of the specimen, and the deformation measured by the extensometer only contains the deformation of the specimen, so the use of the extensometer can more accurately characterize the deformation of the specimen in the experimental process. In Figure 3c, the time-dependent variation in strain is presented, as measured by two strain gauges on the outer surface of the confining cylinder. The nearly identical strain values from different positions affirm the uniformity of the specimen’s deformation. Figure 3d,e depicts the time-dependent variations in circumferential and radial stress–strain of the specimen obtained using Equations (7) and (9).

**Figure 4:**
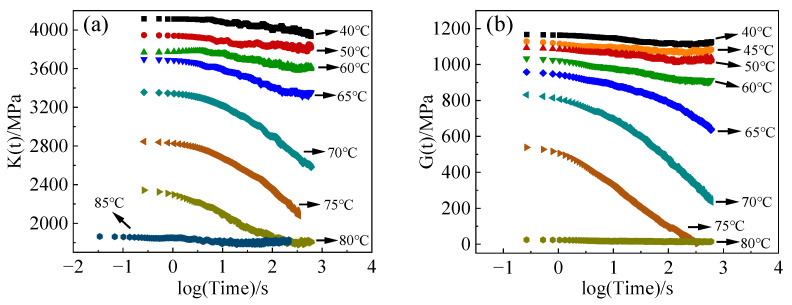
Relaxation curves of specimens at different temperatures: (**a**) bulk modulus relaxation; (**b**) shear modulus relaxation.

The relaxation curves of the bulk and shear modulus of the specimens at different temperatures are summarized as shown in Figure 4. E-44 epoxy resin exhibits strong time and temperature dependence. At low temperatures, the mobility of molecules and chain segments is low, making deformation difficult, and stress relaxation is slower with a smaller amplitude. Conversely, at high temperatures, the mobility of molecules and chain segments is enhanced, and polymer chain segments are more flexible and easy to deform, leading to more significant stress relaxation. Once the temperature surpasses the glass transition temperature, the molecular deformation ability further increases. Consequently, the stress relaxation time decreases dramatically, allowing the specimen to achieve a state of complete relaxation within a short period.

### 4.3. Relaxation Modulus Principal Curves

**Figure 5:**
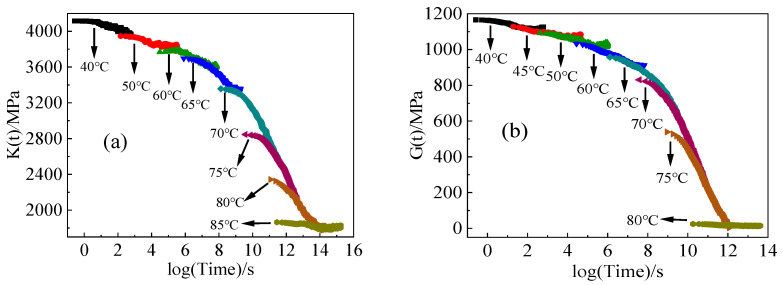
Horizontal translational relaxation modulus curves: (**a**) bulk modulus relaxation curve; (**b**) shear relaxation modulus curve.

The time-temperature superposition (TTS) principle is the foundation for deriving long-term master curves for viscoelastic properties [30]. At low temperatures, the polymer chain segments exhibit high internal friction, resulting in slow and less easily detectable stress relaxation over short durations. Based on this principle, a series of higher temperatures are used to simulate accelerated tests, serving as a substitute for longer relaxation testing times [31]. The results of short-term relaxation tests at different temperatures are considered as part of a master curve shifted parallel to the logarithmic time axis [32]. Using 40 °C as the reference temperature, the bulk and shear relaxation modulus curves of specimens at different temperatures were horizontally shifted on the logarithmic time axis, ensuring that the curves were superimposed and seamlessly connected to each other, as illustrated in Figure 5.

The relaxation modulus curves at 40 °C require no shift and $\mathrm{lg}\alpha_{T}$ is zero. For curves at different temperatures, the relaxation curves shift to the right, with $\mathrm{lg}\alpha_{T}$ being negative. The shifting factors for the relaxation modulus curves at various temperatures are recorded, and the results are presented in Table 4. From the table, it can be concluded that although the shifting factors for the main curves of bulk and shear relaxation modulus are slightly different, the overall trend is the same, increasing with the temperature.

**Figure 6:**
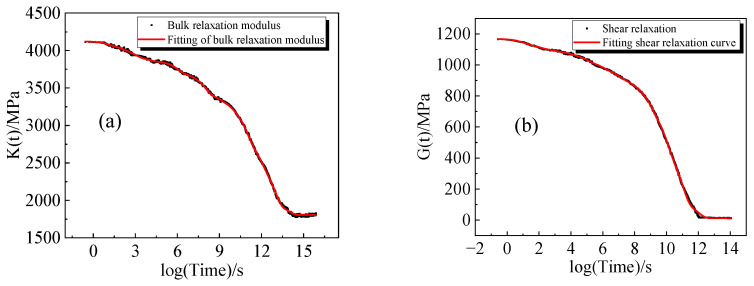
Nonlinear fits: (**a**) bulk relaxation modulus master curve and fit; (**b**) shear relaxation modulus master curve and fit.

To construct the isochronous curves of bulk and shear relaxation modulus with respect to temperature, the relaxation curves were further processed to obtain a continuous master curve for bulk and shear moduli relaxation, as illustrated in Figure 6. Over time, the primary curves of bulk and shear relaxation modulus gradually approach a horizontal trend, ultimately relaxing to the equilibrium state.

### 4.4. Main Curve Fitting

The viscoelastic behavior of epoxy resin can be effectively characterized by the summation of Maxwell elements. Given the time-temperature dependence of its bulk modulus and shear modulus, each element is characterized by a distinctive relaxation time. Employing a viscoelastic constitutive model, the primary curves of bulk and shear relaxation moduli for the specimen are fitted [33]. The fitting formula is expressed as follows:

(15) $$K(\xi )=K^{\infty }+[K^{u}-K^{\infty }]\sum_{\omega =1}^{n}W_{\omega }\exp[\frac{-\xi (T)}{\tau_{\omega }}]$$

(16) $$G(\xi )=G^{\infty }+[G^{u}-G^{\infty }]\sum_{\omega =1}^{n}W_{\omega }\exp[\frac{-\xi (T)}{\tau_{\omega }}]$$

where $K^{\infty }$ and $G^{\infty }$ are the bulk and shear moduli relaxed to equilibrium, $K^{u}$ and $G^{u}$ are the unrelaxed bulk and shear moduli, respectively, ξ(T) is the equivalent time, and $W_{\omega }$ and $\tau_{\omega }$ represent the respective weighting factors and discrete stress relaxation times for the corresponding Maxwell unit.

The fitting results are shown in Figure 6, with R^2^ > 0.99 indicating a high degree of curve fitting accuracy. Table 5 presents the values of the model parameters $K^{\infty }$, $K^{u}$, $G^{\infty }$, and $G^{u}$ obtained through the fitting process.

### 4.5. Experimental Validation

In the context of linear viscoelastic behavior of materials, any analytical work can derive the other functions by describing any two of the four material functions (relaxation or creep) of uniaxial, shear, bulk, or Poisson’s ratio response [34]. The experimentally measured bulk and shear relaxation moduli can be converted to tensile relaxation moduli using the theoretical equation as follows:

(17) $$E(t)=\frac{9K(t)G(t)}{3K(t)+G(t)}$$

We poured and cured the epoxy resin in a glass mold to obtain a 3 mm thick, smooth and flat epoxy resin sheet. We employed a carving machine (Jingxun CNC, QL-1325, Jinan, China) to produce specimens of dimensions 150 × 50 × 3 mm (length, width, height). We performed uniaxial relaxation tests on the specimens to obtain the tensile relaxation modulus master curve. Then, we compared this curve with the tensile relaxation master curve obtained through formula conversion to validate the reliability of the measured bulk and shear relaxation moduli of epoxy resin obtained from this experimental setup.

We converted the testing machine into tensile testing mode to assess the tensile relaxation modulus of epoxy resin at various temperatures. Initially, we secured the lower end of the specimen in a fixture, set the testing temperature, and clamped the upper end of the specimen after the specimen temperature stabilizes. We applied a load to the specimen at a rate of 2 mm/min, characterizing strain during the tensile process using strain gauges affixed at the center of the specimen. Once the load reached 900 N, we left the clamping end position unchanged and recorded the changes in force F and strain ε over time. We calculated the uniaxial relaxation modulus of epoxy resin using the following formula:

(18) $$E(t)=\frac{F(t)}{A\varepsilon (t)}$$

where A is the force area of the specimen.

Similarly, based on the time-temperature superposition principle and using 40 °C as the reference, the relaxation modulus curves at other temperatures were horizontally shifted to obtain the master curve for tensile relaxation modulus and the corresponding shift factors.

**Figure 7:**
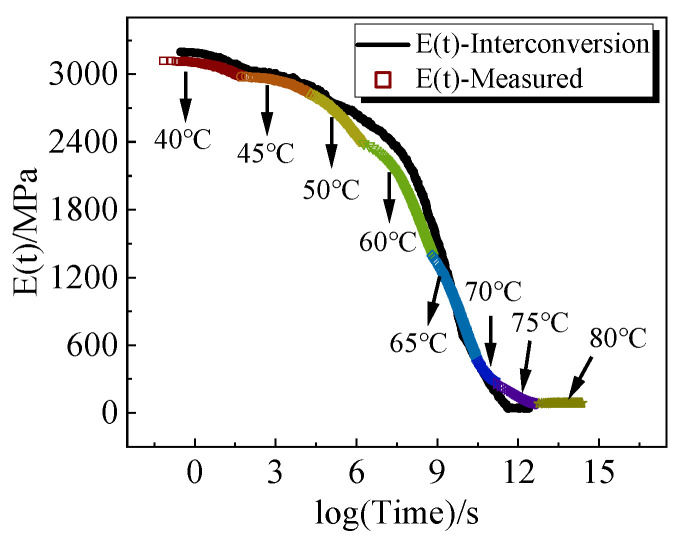
Comparison of the tensile relaxation master curves obtained from uniaxial tensile relaxation experiments with the results obtained by interconverting.

**Figure 8:**
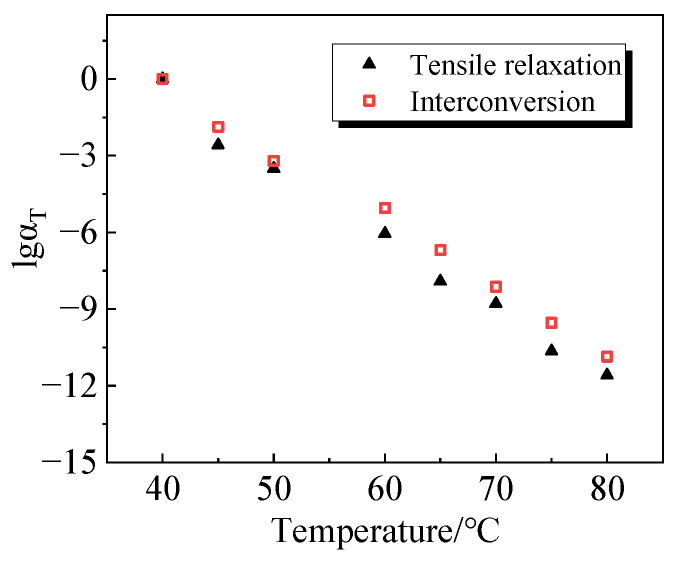
Shift factor of uniaxial relaxation modulus principal curves obtained by two methods.

The tensile relaxation modulus principal curves, derived by *K-G* using Equation (17), are compared with the tensile relaxation modulus principal curves obtained from the experimental test. The results are shown in Figure 7, which shows a good match between the two. The shifting factors of the master curves obtained by the two methods are compared as shown in Figure 8. From the figure, it is evident that the shift factors of the two are remarkably similar, affirming the reliability of the bulk and shear relaxation modulus principal curves obtained through the self-designed molds in this study.

**Figure 9:**
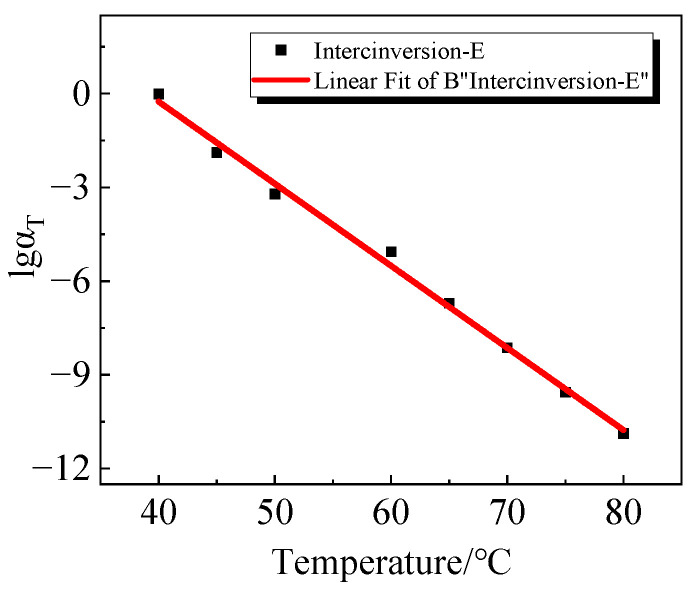
Linear fit to the shift factor.

To illustrate the relationship between the shifting factors of uniaxial relaxation mod-ulus principal curves obtained through *K-G* conversion and temperature, a scatter plot was generated, followed by a linear regression analysis. The outcomes of this analysis are presented in Figure 9, and the mathematical expression describing the correlation between shifting factors and temperature is depicted below:

(19) $$\mathrm{lg}\alpha_{T}=10.2608T-0.2628$$

This equation can be used to derive the displacement factor at different temperatures and to describe the stress–strain behavior of viscoelastic polymers at different temperatures using the time-temperature equivalence principle.

## 5. Conclusions

To investigate the linear viscoelastic properties of potted epoxy resins, a specialized confining pressure testing device was designed. A series of compression tests were conducted on fully cured epoxy resins across various temperatures. Through meticulous data processing, a set of viscoelastic models for bulk and shear moduli were derived. The experimental setup was further validated for reliability using the viscoelastic model obtained from uniaxial tensile tests.

1. A simple method for obtaining the bulk and shear moduli of epoxy resin is described. After applying a load to the experimental setup, a stainless steel tube and cylindrical metal blocks conformed to the specimen, creating confining pressure. Measurements were then taken for the circumferential strain on the outer surface of the cylinder and axial stress–strain of the specimen. The initial bulk and shear moduli of the specimen were deduced by theoretical formulas at different temperatures.
2. We characterized the bulk and shear relaxation modulus of the epoxy resin across a range of eight temperatures, spanning both above and below its glass transition temperature. Subsequently, we derived the relaxation modulus master curve by applying translation to the relaxation modulus curves at different temperatures, adhering to the time-temperature superposition principle.
3. The relaxation modulus master curve is nonlinearly fitted with a viscoelastic principal model to obtain the parameters $K^{\infty }$, $K^{u}$, $G^{\infty }$, and $G^{u}$.
4. The data reliability of the relaxation curves of bulk and shear modulus obtained from the confining pressure test of the homemade mold in this paper was verified by the uniaxial relaxation test.

## Figures and Tables

**Table 1:** Material viscoelasticity test temperature.

**Number**	1	2	3	4	5	6	7	8	9
**Temperature/**°C	40	45	50	60	65	70	75	80	85

**Table 2:** Specification of the confining cylinder.

Number	Inside Diameter/mm	Wall Thickness/mm	Amount
1	16.00	2	3
2	16.02	2	3
3	16.05	2	3
4	16.10	2	3
5	16.12	2	3

**Table 3:** Bulk modulus and shear modulus of materials at different temperatures.

Temperature/°C	Bulk Modulus/MPa	Shear Modulus/MPa
40	4140	1205
45	4101	1160
50	4063	1133
60	3816	1034
65	3769	966
70	3474	857
75	2882	578
80	2387	31
85	1862	19

**Table 4:** Shift factors of bulk and shear modulus relaxation curves of specimens at different temperatures.

Temperature/°C	$lg\alpha_{KT}$	$lg\alpha_{GT}$
40	0	0
45		−1.871
50	−2.9458	−3.206
60	−5.0198	−5.026
65	−6.473	−6.6975
70	−8.992	−8.155
75	−10.4671	−9.5586
80	−11.9096	−11.2634
85	−13.5383	

Note: $\alpha_{KT}$ is the shift factor of the bulk relaxation modulus, and $\alpha_{GT}$ is the shift factor of the shear relaxation modulus.

**Table 5:** Bulk and shear relaxation master curve fitting results.

**Model Parameter**	$K^{\infty }$	$K^{u}$	$G^{\infty }$	$G^{u}$
**Fitting results/MPa**	1805	4122	12	1168

## Data Availability

Data are contained within the article.

## References

[1] Wen Y., Chen C., Ye Y., Xue Z., Liu H., Zhou X., Zhang Y., Li D., Xie X., Mai Y. (2022). Advances on Thermally Conductive Epoxy-Based Composites as Electronic Packaging Underfill Materials—A Review. Adv. Mater..

[2] Cheah L.B., Heng C.W., Ng S.Y., Teh P.L., Poopalan P. (2020). UV Modified Epoxy for LED Encapsulant. J. Phys. Conf. Ser..

[3] Zhang C., Liu Z., Zhang T., Wang X., Yin C., Liu X., Chi Q. (2023). High-temperature resistance and excellent electrical insulation in epoxy resin blends. J. Appl. Polym. Sci..

[4] Sun W., Wang L., Zhu N., Xin J., Luo Y., Jiang X., Fan G., Chen M. (2023). Characterization of Packaging Warpage, Residual Stress and Their Effects on the Mechanical Reliability of IGBT Power Modules. Eng. Fail. Anal..

[5] Huskić M., Kusić D., Pulko I., Nardin B. (2022). Determination of residual stresses in amorphous thermoplastic polymers by DMA. J. Appl. Polym. Sci..

[6] Courtois A. (2019). Viscoelastic behavior of an epoxy resin during cure below the glass transition temperature: Characterization and modeling. J. Compos. Mater..

[7] Politi M., Breuer O., Cohen Y. (2023). Simulation and Experimental Validation of the Cure Process of an Epoxy-Based Encapsulant. Exp. Mech..

[8] Qu C., Zhang X., Wang D., Fan X., Li H., Liu C., Feng H., Wang R., Guo K., Tian Y. (2022). Residual stress and thermal properties of rubber-modified epoxy systems for semiconductor package. J. Appl. Polym. Sci..

[9] Namazian Z., Yaghoubi M. (2019). Optimization of thermal curing cycle for a large epoxy model. J. Comput. Appl. Mech..

[10] Nunes S.G., Saseendran S., Joffe R., Amico S.C., Fernberg P., Varna J. (2020). On temperature-related shift factors and master curves in viscoelastic constitutive models for thermoset polymers. Mech. Compos. Mater..

[11] Avila Torrado M., Constantinescu A., Johlitz M., Lion A. (2022). Viscoelastic behavior of filled silicone elastomers and influence of aging in inert and hermetic environment. Contin. Mech. Thermodyn..

[12] Saseendran S., Wysocki M., Varna J. (2017). Evolution of viscoelastic behaviour of a curing LY5052 epoxy resin in the rubbery state. Adv. Compos. Mater..

[13] Agha A., Abu-Farha F. (2022). Numerical implementation and validation of a viscoelastic-plastic material model for predicting curing induced residual stresses in adhesive bonded joints. Int. J. Adhes. Adhes..

[14] Adrover-Monserrat B., García-Vilana S., Sánchez-Molina D., Llumà J., Jerez-Mesa R., Travieso-Rodriguez J.A. (2022). Viscoelastic Characterization of a Thermoplastic Elastomer Processed through Material Extrusion. Polymers.

[15] Nawab Y., Casari P., Boyard N., Jacquemin F. (2013). Characterization of the cure shrinkage, reaction kinetics, bulk modulus and thermal conductivity of thermoset resin from a single experiment. J. Mater. Science..

[16] Sangtabi M.R., Kiasat M.S. (2017). Long-term viscoelastic properties of an adhesive and molding compound, characterization and modeling. Polymer.

[17] Müller-Pabel M., Agudo J.A.R., Gude M. (2022). Measuring and understanding cure-dependent viscoelastic properties of epoxy resin: A review. Polym. Test..

[18] Klimm W., Kwok K. (2022). Mechanism of resistance relaxation and hysteresis in viscoelastic piezoresistive polymer nanocomposites. Int. J. Mech. Mater. Des..

[19] Ikeda T., Kanno T., Shishido N., Miyazaki N., Tanaka H., Hatao T. (2013). Non-linear analyses of strain in flip chip packages improved by the measurement using the digital image correlation method. Microelectron. Reliab..

[20] Vieira de Mattos D.F., Huang R., Liechti K.M. (2020). The effect of moisture on the nonlinearly viscoelastic behavior of an epoxy. Mech. Time-Depend. Mater..

[21] Sfar Zbed R., Le Corre S., Sobotka V. (2022). Process-induced strains measurements through a multi-axial characterization during the entire curing cycle of an interlayer toughened Carbon/Epoxy prepreg. Compos. Part A Appl. Sci. Manuf..

[22] Qamar S.Z., Akhtar M., Pervez T., Al-Kharusi M.S.M. (2013). Mechanical and structural behavior of a swelling elastomer under compressive loading. Mater. Des..

[23] Barney C.W., Helgeson M.E., Valentine M.T. (2022). Network structure influences bulk modulus of nearly incompressible filled silicone elastomers. Extrem. Mech. Lett..

[24] Senol K., Shukla A. (2019). Underwater mechanical behavior of closed cell PVC foams under hydrostatic loading through 3D DIC technique. Polymer. Test..

[25] Lee H.S., Sun Y., Kim C., Han B. (2018). Characterization of linear viscoelastic behavior of epoxy molding compound subjected to uniaxial compression and hydrostatic pressure. IEEE Trans. Compon. Packag. Manuf. Technol..

[26] Ravi-Chandar K., Ma Z. (2000). Inelastic Deformation in Polymers under Multiaxial Compression. Mech. Time-Depend. Mater..

[27] Qvale D., Ravi-Chandar K. (2004). Viscoelastic Characterization of Polymers under Multiaxial Compression. Mech. Time-Depend. Mater..

[28] Li S., Sitnikova E., Liang Y., Kaddour A.-S. (2017). The Tsai-Wu failure criterion rationalised in the context of UD composites. Compos. Part A Appl. Sci. Manuf..

[29] Kabir H., Aghdam M.M. (2019). A robust Bézier based solution for nonlinear vibration and post-buckling of random checkerboard graphene nano-platelets reinforced composite beams. Compos. Struct..

[30] Henriques I.R., Rouleau L., Castello D.A., Borges L., Deü J.-F. (2020). Viscoelastic behavior of polymeric foams: Experiments and modeling. Mech. Mater..

[31] Schalnat J., Daelemans L., De Baere I., De Clerck K., Van Paepegem W. (2021). Long-term stiffness prediction of particle filled polymers by dynamic mechanical analysis: Frequency sweep versus creep method. Polym. Test..

[32] Phansalkar S.P., Kim C., Han B. (2022). Effect of critical properties of epoxy molding compound on warpage prediction: A critical review. Microelectron. Reliab..

[33] Prasatya P., McKenna G.B., Simon S.L. (2001). A Viscoelastic Model for Predicting Isotropic Residual Stresses in Thermosetting Materials: Effects of Processing Parameters. J. Compos. Mater..

[34] Tschoegl N.W., Knauss W.G., Emri I. (2002). Poisson’s Ratio in Linear Viscoelasticity—A Critical Review. J. Mech. Time-Depend. Mater..

